# Shorter Anogenital Distance in Women with Ovarian Endometriomas and Adenomyosis, but Not Uterine Leiomyomas

**DOI:** 10.3390/biomedicines11102618

**Published:** 2023-09-23

**Authors:** Xishi Liu, Ding Ding, Minhong Shen, Dingmin Yan, Sun-Wei Guo

**Affiliations:** 1 Department of Gynecology, Shanghai Obstetrics and Gynecology Hospital, Fudan University, Shanghai 200011, China; lxsdoc@hotmail.com (X.L.); dingdinggyn1208@163.com (D.D.); minhongshen0128@163.com (M.S.); ydm_og@126.com (D.Y.); 2 Shanghai Key Laboratory of Female Reproductive Endocrine-Related Diseases, Fudan University, Shanghai 200011, China; 3 Research Institute, Shanghai Obstetrics and Gynecology Hospital, Fudan University, Shanghai 200011, China

**Keywords:** adenomyosis, anogenital distance, digit ratio, endometriosis, estrogen, prenatal exposure, uterine leiomyomas

## Abstract

We investigated whether anogenital distance (AGD) is associated with adenomyosis, endometriosis and uterine leiomyomas (UL, also called uterine fibroids). We recruited 81 women with UL, 105 with ovarian endometrioma (OE), 116 with adenomyosis, 28 with both adenomyosis and UL, and 100 control subjects with other acquired gynecological conditions but not endometriosis, adenomyosis, UL, or polycystic ovarian syndrome. Measurements from the anterior clitoral surface to the center of the anus (AGD_AC_), from the tip of the clitoris to the center of the anus (AGD_ACt_), and from the posterior fourchette to the center of the anus (AGD_AF_) were made in all subjects. Multiple regression was performed to estimate the association between AGDs and presence of OE, adenomyosis, and UL while controlling for possible confounding factors. We found that, compared with controls, women with OE and adenomyosis, but not UL, had significantly shorter AGD_AF_, but not AGD_AC_. However, the amount of variance that could be explained by the disease status is rather moderate, suggesting that factors other than disease status, bodyweight and height were also responsible for AGD. Thus, prenatal exposure to reduced levels of androgen may increase the risk of developing endometriosis and adenomyosis. However, other factors may also contribute to the pathogenesis of endometriosis and adenomyosis.

## 1. Introduction

Uterine leiomyomas (also known as uterine fibroids, or myomas) are the most common benign tumors of the uterus with an estimated prevalence of over 75% of women worldwide [1,2]. Next to uterine leiomyomas (UL), endometriosis and adenomyosis are two of the most common gynecological disorders worldwide, affecting 6–10% [3] and approximately 20% [4], respectively, of women of reproductive age. The three diseases cause similar and overlapping symptoms, ranging from pelvic pain, infertility, and menstrual disturbances [5]. In particular, UL and adenomyosis are known to be two structural causes of abnormal uterine bleeding [6], especially heavy menstrual bleeding. The pathogeneses of all three most common gynecological diseases are poorly understood [7,8,9,10,11], and, as such, the three diseases are often viewed as “enigmatic” [10,12,13]. The enigma is further shrouded in riddles and conundrums since these diseases often co-exist [14,15,16]. 

Defined as the distance between the anus and the genital tubercle, anogenital distance (AGD) is a sexually dimorphic feature [17,18]. In utero androgen levels affect the development of the perineal tissue and are negatively correlated with the AGD [19], which can serve as a life-long indicator of androgen, vis-à-vis estrogen, action in gestational weeks 8–14, which are known as the masculinization programming window [20]. Hence, AGD represents a biomarker of the prenatal hormonal environment [21,22], although AGD also can reflect the prenatal exposure to endocrine disruptors [23,24] and androgens during the development of the reproductive system [25,26]. 

Two AGD measurements in females are commonly used in the literature. One is AGD_AC_, defined to be the distance from the anterior clitoral surface to the upper/center verge of the anus, and the other, AGD_AF_, is defined as the distance from the posterior fourchette to the upper/center verge of the anus [17,27,28,29,30].

A shorter AGD, especially AGD_AF_, has been reported to be associated with a higher risk of endometriosis, especially deep endometriosis [17,27,28,29,30]. In contrast, longer AGD is reported to be associated with polycystic ovarian syndrome (PCOS) [31,32,33,34,35]. This contrasting difference in AGD, presumably attributable to a difference in hormonal environment in utero, between endometriosis and PCOS, led to the proposal that these two diseases represent extreme and diametric (opposite) outcomes of variation in the hypothalamic–pituitary–gonadal axis development and activity [36], potentially with a far-reaching evolutionary significance [37].

Given the reported diametric distribution in AGD between endometriosis and PCOS, one may wonder whether adenomyosis and UL are also associated with shorter AGD since these two conditions are also known to be estrogen-dependent, as in endometriosis. In addition, several studies reported that AGD_AF_ performs better than AGD_AC_ in separating endometriosis and controls [29], and in one study AGD_AC_ was simply forfeited [28]. It is speculated that this may be due to the differences in the size of the fat pad anterior to the pubic *symphysis*, an area that is included in AGD_AC_ but not in AGD_AF_ [29]. This raises a question as to whether an AGD measurement, based on certain landmark that is between the anterior clitoral surface and the posterior fourchette, would perform better than AGD_AC_ but probably no better than AGD_AF_. 

Moreover, a recent well-designed case–control study failed to replicate the association between AGD and endometriosis [38]. This calls for more investigation into the relationship. 

While most, if not all, studies on the relationship between AGD and endometriosis/PCOS focused on the discriminative capability of AGD, it is perhaps self-evident that, aside from intrauterine hormonal environment, many other factors may contribute to the genesis and development of the diseases. For adenomyosis, in particular, iatrogenic uterine procedures have been consistently reported to increase the risk of developing adenomyosis [39,40,41,42,43,44], which has been recently demonstrated experimentally [45]. Therefore, instead of constructing an AGD-based diagnostic criterion, it is perhaps more appropriate to assess just how much variation in AGD measurements the disease status can account for. This becomes especially relevant since in clinical settings co-morbidity among endometriosis, adenomyosis, and UL is quite common [14,15,16]. 

In this study, we investigated the relationship between AGD and the three common gynecological diseases, endometriosis, adenomyosis, and UL based on a total of 430 patients, controlling for other possible confounders such as age, height, weight, number of vaginal deliveries, and history of episiotomy. In view of frequent discordant findings using two popular AGD measurements AGD_AC_ and AGD_AF_, we also came up with a third AGD measurement, called AGD_ACt_, defined as the distance from the tip of the clitoris to the center of the anus, which was strategically designed to vary between AGD_AC_ and AGD_AF_ so that we could use it to test the hypothesis that AGD_AC_ is less reliable than AGD_AF_ because the former measurement encompasses two sources of variation: variation in genuine AGD and variation in the development of genitalia. Moreover, since the ratio between the second and fourth digits (2D:4D ratio) is hypothesized to be a proxy of prenatal exposure to sex hormones and might affect the risk of hormone-related diseases [46,47] such as endometriosis [30], we also investigated the relationship between the digit ratios and the three diseases. We found that both endometriosis and adenomyosis, but not UL, are associated with a shorter AGD. However, the amount of variance that can be explained by the disease status is quite moderate. Digit ratios were not found to be associated with any of these diseases.

## 2. Materials and Methods

### 2.1. Patient Recruitment and Data Collection

This study, conducted from January to August 2021 at the Department of Gynecology, Shanghai Obstetrics and Gynecology Hospital, Fudan University, Shanghai, China, was approved by the ethical committee of Shanghai Obstetrics and Gynecology Hospital, Fudan University (No. 2020-35). A total of 430 premenopausal patients were recruited into this study after informed consent, and were grouped as controls, endometriosis, adenomyosis, UL, and mixed. The sample size was determined based on previous publications on the association between AGD and endometriosis. 

The group designation was based primarily on signs and symptomology, gynecological examination, imaging findings, and surgical indications, especially for patients with endometriosis and a co-occurrence of adenomyosis. For example, if the main symptoms were dysmenorrhea or heavy menstrual bleeding with or without anemia, along with an enlarged uterus of over 6 weeks of gestation, and at the same time, the OE was less than 4 cm, we classified this kind of cases into the adenomyosis group. On the other hand, if the main surgical indication was an OE that was larger than 4 cm, a DE greater than 2 cm or a DE with resulting obstructive symptoms, while the size of the uterus was less than 6 weeks of gestation, we classified this patient as in the endometriosis group. In particular, in three cases where endometriosis coexisted with adenomyosis, their OE lesions were larger than 5 cm, and their uterine sizes were larger than 6 weeks of gestation; as such, they were classified into the endometriosis group because the surgical indication was OE. Endometriosis and adenomyosis patients who were coexisting with UL were classified into endometriosis or adenomyosis group according to the above criteria, irrespective of their UL size. We note that, in clinical settings, many patients have co-morbidities and, as such, our grouping merely reflected the clinical reality. More importantly, we note that when it comes to data analysis, we did not analyze the difference purely based on grouping but rather on group indicators since a patient could have OE, DE, UL, and/or adenomyosis or a combination of these diseases. Thus, we could take all these comorbidity and multiple groupings into account, and fully use the data.

Among the recruited patients, 81 cases were diagnosed with UL only (UL group), 105 cases were diagnosed with endometriosis (including ovarian endometrioma (OE), deep endometriosis (DE), peritoneal endometriosis, and abdominal wall endometriosis; the endometriosis group), 116 cases were diagnosed with adenomyosis (adenomyosis), and 28 cases were diagnosed with adenomyosis coexisting with UL (mixed), and 100 patients served as controls (control). All diagnoses were made based on symptoms, gynecological examinations, ultrasonography, and magnetic resonance imaging (MRI). All patients underwent transvaginal ultrasonography (TVUS) examination. 

Among these patients, 7 (8.6%), 50 (47.6%), 73 (62.9%), 15 (53.5%), and 14 (14.0%) patients in the UL, endometriosis, adenomyosis, mixed, and control groups also underwent MRI examinations, respectively. Among the controls, 24 had vaginitis; 22 were healthy women who visited the health examination clinic; 12 had cervical cancer in situ, or high- or low-grade squamous intraepithelial lesion (HSIL or LSIL); 10 had stage I/II cervical cancer; 10 had human papilloma virus infection and cervicitis; 7 had salpingitis or hydrosalpinx; 6 each had ovarian teratoma and Mullerian’s duct cysts of the fallopian tubes; and 3 had serous ovarian cyst. No endometriosis, adenomyosis, UL, or PCOS was detected via TVUS and/or MRI and, in some patients, surgical exploration, nor were an associated symptoms and surgical history for these diseases found in the controls. 

The criteria for the TVUS diagnosis of OE or DE were as follows: (1) routine evaluation of the presence or absence of endometriotic cysts; (2) TVUS evaluation of “soft markers” such as site-specific tenderness and ovarian mobility; (3) assessment of status of pouch of Douglas using the real-time ultrasound-based “sliding sign”; and (4) assessment of DE nodules in the anterior and posterior compartments, which involved the assessment of the bladder, vaginal vault, uterosacral ligaments, and bowel, including the rectum or rectosigmoid junction [48,49]. The lesions of the abdominal wall endometriosis were nodules at Cesarean section scar, which had an isoechoic or hyperechoic pattern, with peripheral vascularization under ultrasonography [50].

The TVUS diagnosis of adenomyosis was based on the globally enlarged uterus, the lesional echo and site, the thickness of the myometrium, and the thickness of the junctional zone. The echogenicity of the lesion was compared to that of the adjacent myometrium, and there was no obvious boundary between the lesion and the adjacent myometrium. Hyperechogenic islands were lesional areas within the myometrium and could be regular or irregular. Sometimes, the myometrial cysts could be seen within the myometrium [51,52].

The TVUS diagnosis of UL was based on the characteristic findings of an irregularly enlarged uterus and the hypoechoic nodule surrounded by pseudocapsule, which presented as a white ring surrounding the UL and was often colored as a ‘‘ring of fire’’ by Doppler scans, surrounding and separating the UL from normal myometrium [53].

In addition, the demographic and clinical information of all patients, such as age, age at menarche, birth weight (in kg), gravidity, parity, verbal rating scale on the severity of dysmenorrhea, number of vaginal deliveries if any, history of episiotomy, history of Cesarean section and of intrauterine surgery were collected. Moreover, height (in meters) and bodyweight (in kg) were also measured and then their body mass index (BMI) calculated.

### 2.2. Measurement of Anogenital Distances 

**Figure 1:**
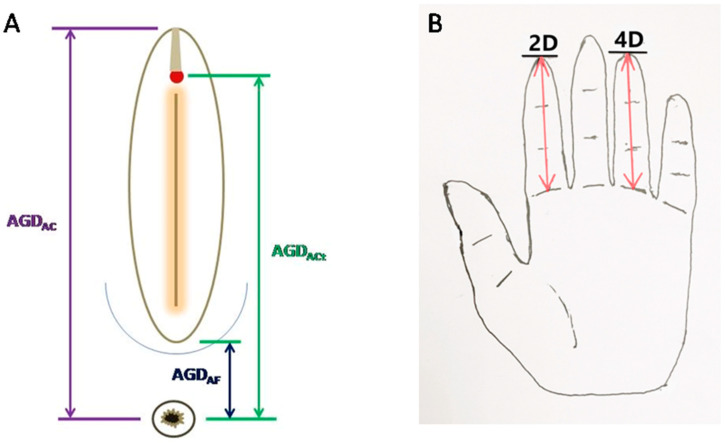
Measurement of anthropometric biomarkers. (**A**) A schematic illustration demonstrating key landmarks for the three measurements of anogenital distance (AGD): AGD_AC_, from the anterior clitoral surface to the center of the anus (left); AGD_AF_, from the posterior fourchette to the center of the anus (middle); and AGD_ACt_, from the tip of the clitoris to the center of the anus (right). (**B**) Ratio between second and fourth digits (2D:4D ratio).

The AGD was measured using a digital caliper with women laying down in the lithotomy position to the examination table with thighs at a 45° angle. For each woman, three AGD measurements were obtained: AGD_AC_, from the anterior clitoral surface (root of the clitoral hood) to the center of the anus, AGD_ACt_, from the tip of the clitoris to the center of the anus, and AGD_AF_, from the posterior fourchette to the center of the anus (Figure 1A). The measurements of AGD_AC_ and AGD_AF_ were identical to those in Peters et al. [30] but slightly different from [17,28,29,38,54] in that we used the center of the anus instead of the upper verge of the anus in order to minimize the variation in the width of the upper verge. The tips of the digital caliper were filed to dull the sharpness. 

To improve precision, two experienced gynecologists that were not directly involved in the clinical evaluation of the patients measured each distance twice, resulting in a total of six measurements of AGD_AC_, AGD_ACt_, and AGD_AF_ for each woman. The average values of the measurements were used as estimates of each AGD measurement. 

### 2.3. Ratio between the Second and Fourth Digits

For the ratio of the length of the second finger by the fourth finger (2D:4D), we used the direct measurement a digital caliper, similar to the one used for AGD measurement. We measured the length of the second and the fourth fingers for both left and right hands. Following Peters et al. [30], the digit lengths were measured on the ventral surface of the hand, from the basal crease of the digit to the tip of the finger in the midline (Figure 1B). The digit ratio was calculated by dividing the length of the second finger by the length of the fourth finger. To minimize measurement errors, two persons who performed the AGD measurements carried out the 2D:4D measurements, twice, and the average values were used for analysis. 

### 2.4. Statistical Analysis

Fisher’s exact test was used to compare the contingency table data between two groups. The comparison of distributions of continuous variables between or among two groups was made using Wilcoxon’s test. Pearson’s correlation coefficient was used to calculate the correlation between two variables. Multivariate linear regression analyses were used to determine which factors were associated with AGD measurements and digit ratios when accounting for possible confounders such as age, parity, BMI, episiotomy, and co-morbidity. *p*-values of < 0.05 were considered statistically significant. All computations were made with R 4.2.2 [55].

## 3. Results

### 3.1. Basic Characteristics

The characteristics of all recruited patients are shown in Table 1. We can see that the patients in the endometriosis group were mostly those with OE (95.2%), and the remaining patients had abdominal wall endometriosis (two cases), and DE only (two cases, one each of bladder endometriosis and sacral ligament, DE), and perineal endometriosis (one case). Patients in the mixed group all had both adenomyosis and uterine leiomyomas. Patients from the endometriosis group were comparable in age with the control group, but those with adenomyosis, UL, or mixed conditions were significantly older than that of control group (Supplementary Figure S1). All groups had comparable height, but patients in the UL and the mixed groups were significantly heavier in bodyweight as compared with the control group (Table 1 and Supplementary Figure S1). Endometriosis patients also were comparable in BMI with the control group, but those with adenomyosis, UL, or mixed conditions had a significantly higher BMI than the control group (Table 1 and Supplementary Figure S1). Since only one patient each smoked (control group) or drank (Adenomyosis group), neither smoking nor drinking status was considered in our analysis. 

All groups of patients were comparable in age at menarche, birth weight, frequency of vaginal deliveries, and episiotomy, but patients in the adenomyosis group had significantly higher parity than the control group (Table 1 and Supplementary Figure S1). Patients from both endometriosis and adenomyosis groups had significantly more Cesarean sections. In fact, among all recruited patients, those who had at least one Cesarean section were associated with a significantly higher risk of adenomyosis (odds ratio (OR) = 1.93, 95% confidence interval (CI) = 1.26–2.97), but not endometriosis (OR = 1.21, 95% CI = 0.76–1.92). Furthermore, those with at least one uterine procedure had a significantly higher risk of adenomyosis (OR = 2.34, 95% CI = 1.50–3.71), but not endometriosis (OR = 0.65, 95% CI = 0.42–1.03). 

### 3.2. Anogenital Distance Measures

**Figure 2:**
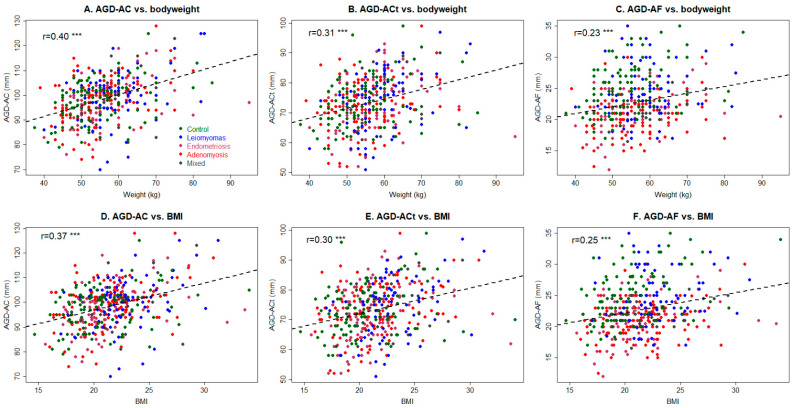
The relationship between different AGD measurements and bodyweight and body mass index (BMI). Scatter plots showing the relationship between bodyweight and AGD_AC_ (**A**), AGD_ACt_ (**B**) and AGD_AF_ (**C**), and between BMI and AGD_AC_ (**D**), AGD_ACt_ (**E**), and AGD_AF_ (**F**). Notice that the linearity appears to be less and less evident (as the data become more and more scattered) as the point of the measurement became closer to the anus. Each dot represents one data point, and the dashed line represents the regression line. Pearson’s correlation coefficient, along with its statistical significance level, is shown in each plot. Symbol for statistical significance level: ***: *p* < 0.001. AM: adenomyosis; EM: endometriosis; MX: mixed; UL: uterine leiomyomas.

The three AGD measurements were mutually and positively correlated (r = 0.74, *p* < 2.2 × 10^−16^, between AGD_AC_ and AGD_Act_; r = 0.33, *p* = 2.9 × 10^−12^, between AGD_AC_ and AGD_AF_; and r = 0.42, *p* < 2.2 × 10^−16^, between AGD_ACt_ and AGD_AF_), and the correlation coefficient was larger when the two points of measurements were adjacent, which makes sense. Height was positively correlated with AGD_AC_ (r = 0.13, *p* = 0.009), but not AGD_ACt_ (r = 0.06, *p* = 0.19) or AGD_AF_ (*p* = 0.02, *p* = 0.76). The body weight was positively correlated with all AGD measures, but the correlation coefficient became progressively smaller as the point of measurement went lower (r = 0.40, *p* < 2.2 × 10^−16^, r = 0.31, *p* = 7.3 × 10^−11^, and r = 0.23, *p* = 1.7 × 10^−6^; Figure 2A–C). BMI was positively correlated with AGD_AC_, AGD_ACt_, and AGD_AF_ (r = 0.37, *p* = 4.2 × 10^−15^, r = 0.30, *p* = 3.0 × 10^−10^, and r = 0.25, *p* = 4.2 × 10^−7^; Figure 2D–F), with the magnititude of correlation being higher if the point of measurement was higher. Patients with a history of episiotomy had a longer AGD_AC_ (*p* = 0.0498), but no AGD_Act_ or AGD_AF_ (*p* = 0.11 and *p* = 0.88).

### 3.3. AGD Measurements in Different Patient Groups

**Figure 3:**
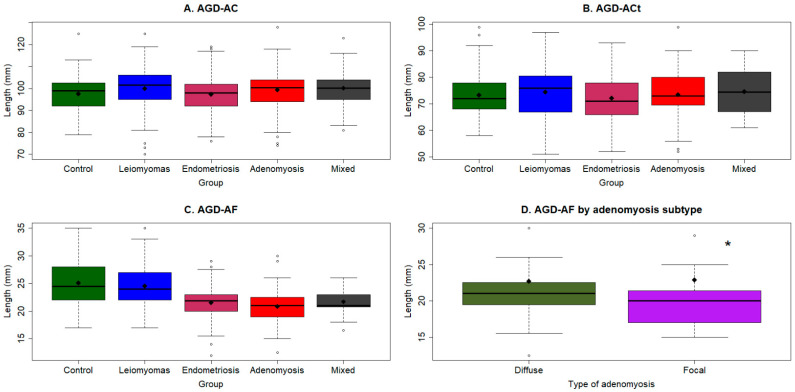
Distributions of different AGD measurements in different patient groups. Each dot represents one data point, with different colors representing different groups. The dashed line represents the regression line. Boxplot showing the distribution of AGD_AC_ (**A**), AGD_ACt_ (**B**), and AGD_AF_ (**C**) in the five patient groups. (**D**) Boxplot showing the distribution of AGD_AF_ within the adenomyosis group. The black diamond in each box represents the mean of the data. Symbol for statistical significance level: *: *p* < 0.05.

Univariate analysis showed that AGD_AC_ in the endometriosis, adenomyosis and mixed groups were comparable with the control group but that in the UL group was significantly longer (Table 2 and Figure 3A). Multiple linear regression analysis incorporating age, age at menarche, parity, number of vaginal deliveries, history of episiotomy, height, bodyweight, presence of OE, pelvic endometriosis, DE, other types of endometriosis, focal or diffuse adenomyosis, and UL as covariables indicates that weight and the history of episiotomy were the only two covariables that were positively associated with AGD_AC_ (*p* < 2.2 × 10^−16^, and *p* = 0.028, respectively; *R*^2^ = 0.17). In other words, only a heavier bodyweight and a history of episiotomy were associated with longer AGD_AC_, but the variance that can be explained by these two variables was quite limited. 

Similarly, all five groups of patients had comparable AGD_ACt_ (Table 2 and Figure 3B). Using the multiple linear regression analysis incorporating age, age at menarche, parity, number of vaginal deliveries, history of episiotomy, height, bodyweight, presence of OE, pelvic endometriosis, DE, other types of endometriosis, focal or diffuse adenomyosis, and UL as covariables, we found, again, that bodyweight and the history of episiotomy were positively associated, while OE was negatively associated with AGD_ACt_ (*p* = 4.9 × 10^−11^, *p* = 0.026, and *p* = 0.020, respectively; *R*^2^ = 0.12). Removing OE or the history of episiotomy both yielded an *R*^2^ value of 0.11, but removing the bodyweight resulted in a *R*^2^ = 0.02, suggesting that the amount of variance in AGD_ACt_ that could be explained by the presence of OE is very limited.

With the exception of the UL group, patients from the endometriosis, adenomyosis, and mixed groups all had a significantly shorter AGD_AF_ as compared with the control group (Table 2 and Figure 3C). Multiple linear regression analysis incorporating age, age at menarche, parity, number of vaginal deliveries, history of episiotomy, height, body weight, presence of OE, pelvic endometriosis, DE, other types of endometriosis, focal or diffuse adenomyosis, and UL as covariables revealed that while bodyweight was positively associated with AGD_AF_ (*p* = 4.1 × 10^−9^), OE and both focal and diffuse adenomyosis, as well as height, were negatively associated with AGD_AF_ (*p* = 4.0 × 10^−8^, *p* = 7.6 × 10^−10^, *p* = 1.6 × 10^−13^, and *p* = 0.014, respectively; *R*^2^ = 0.25). Removing the OE yielded an *R*^2^ of 0.19, while removing both focal and diffuse adenomyosis resulted in an *R*^2^ of 0.16. Among patients with adenomyosis, those with focal adenomyosis had a significantly shorter AGD_AF_ (*p* = 0.022; Figure 3D), but no AGD_AC_ or AGD_ACt_ (*p* = 0.29 and *p* = 0.061). These results suggest that the presence of adenomyosis could explain more variance in AGD_AF_ than the presence of OE.

### 3.4. Digit Ratios (2D:4D)

**Figure 4:**
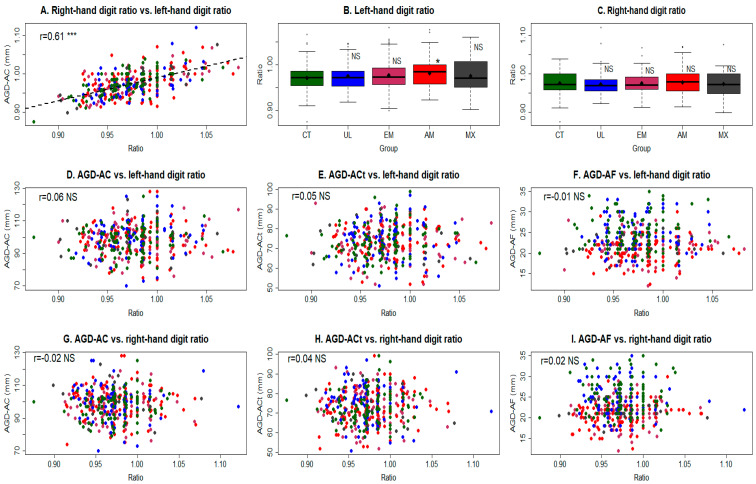
(**A**) Scatter plot showing the relationship between the left-hand digit ratio and the right-hand digit ratio. Boxplot showing the distribution of the left-hand digit ratio (**B**) and right-hand digit ratio (**C**) in the five patient groups. Scatter plots showing the relationship between the left-hand digit ratio and AGD_AC_ (**D**), AGD_ACt_ (**E**), and AGD_AF_ (**F**), and between the right-hand digit ratio and AGD_AC_ (**G**), AGD_ACt_ (**H**), and AGD_AF_ (**I**). Pearson’s correlation coefficient, along with its statistical significance level, is shown in each scatter plot. In (**B**,**C**), the statistical comparison was made between the designated group and the control group, using Wilcoxon’s test. Symbol for statistical significance level: NS: *p* > 0.05; *: *p* < 0.05; ***: *p* < 0.001. Abbreviations for group names: CT: controls; UL: uterine leiomyomas; EM: endometriosis; AM: adenomyosis; MX: mixed.

The left- and right-hand digit ratios were positively correlated (r = 0.61, *p* < 2.2 × 10^−16^; Figure 4A), but neither of them were correlated with BMI or bodyweight (all r’s near 0, all *p*’s > 0.05). With the only exception of the adenomyosis group for the left-hand digit ratio (*p* = 0.037), all other groups had comparable digit ratios with the control group (Table 2; Figure 4B,C). Multiple linear regression analysis incorporating age, age at menarche, parity, number of vaginal deliveries, history of episiotomy, height, bodyweight, presence of OE, pelvic endometriosis, DE, other types of endometriosis, focal or diffuse adenomyosis, and UL as covariables revealed that neither measurement was associated with any covariable. Neither ratio was correlated with any AGD measurement (−0.03 < r < 0.06, all *p*’s > 0.24; Figure 4D–I). Thus, the digit ratios were found to be unrelated with the disease type or status. 

## 4. Discussion

In this study, we have shown that, compared with controls, women with OE and adenomyosis, but not UL, had significantly shorter AGD_AF_, but not AGD_AC_, after controlling for possible confounding factors such as age, age at menarche, parity, height, bodyweight, number of vaginal deliveries, and history of episiotomy. In particular, women with focal adenomyosis had significantly shorter AGD_AF_ than those with diffuse adenomyosis. Consistent with our hypothesis, OE patients, but not adenomyosis patients, had a significantly shorter AGD_ACt_. All AGD measures were positively correlated with the bodyweight, especially when the point of measurement was higher in the body relative to the ground. However, the amount of variance in AGD_AF_ that could be explained by OE and adenomyosis status is rather moderate (ranging from 6% to 15%), suggesting that factors other than the disease status, bodyweight and height are also responsible for AGD_AF_. Finally, digit ratios were unrelated with the disease status. 

Our results are consistent with the previous reports that women with endometriosis had a shorter AGD_AF_ [17,27,28], and are also consistent with the report that prenatal exposure to diethylstilbestrol (DES), a synthetic non-steroid estrogen, resulted in an increased risk of developing endometriosis in adult life [56] and with the report of a possible multigenerational and likely transgenerational effect of fetal exposition to DES [57].

Our results are partially in agreement with the previous finding of a shorter AGD_AC_, but not AGD_AF_, in women with endometriosis [30]. It is likely that the discrepancy may be attributable to the composition of patients with endometriosis since, in Peters et al., only women with DE (infiltrating the peritoneum by 0.5 mm) and/or rASRM stage IV were included, while in our study, nearly 95% of endometriosis patients were OE. Due to the documented link between DE and adenomyosis [58,59], it is possible that those patients purportedly with endometriosis in [30] might also have adenomyosis. In addition, DE and rASRM stage IV endometriosis represent more severe forms of endometriosis, and, as such, may be somewhat different from the other endometriosis patients.

Our results are also consistent with those found in Peters et al. in that that no difference in digit ratio was found among patients with endometriosis, PCOS, Mayer–Rokitansky–Kuster–Hauser syndrome, or the controls [30]. In addition, our finding that the digit ratios were uncorrelated with any of the three AGD measurements is in agreement with a recent report that the digit ratio was not associated with maternal sex steroid concentrations in early pregnancy and AGD in preschool children [60]. We concur with the view expressed in [30] that the digit ratio may represent an insufficient or weak measure reflecting prenatal androgen exposure. 

Our results are at odds with those recently reported by Buggio et al., who found that M_OE_ < M_DE_ < M_CT_ for AGD_AF_ and M_DE_ < M_OE_ < M_CT_ for AGD_AC_, where M denotes the mean, and OE, DE, and CT denote the patients with OE and DE and the controls, respectively, but the difference did not reach statistical significance [38]. Interestingly, for both AGD_AF_ and AGD_AC_ in that study, the two measurements from patients with endometriosis as a whole were 6.1% and 2.2% shorter than those of the controls, and, not surprisingly, the difference was the larger for AGD_AF_. While the sample sizes were calculated based on the largest study on AGD–endometriosis [17], they may be nonetheless inadequate since the actual standard deviation (SD) encountered in Buggio et al. is substantially larger than the assumed one (7.8 vs. 6). Based on their actual data (mean = 23.7, SD = 7.8), and the mean reduction of 2, the sample sizes under type I and type II errors of 0.025 and 0.20 would be much larger than 135, as used in this study. In other words, the study of Buggio et al. [38] might have been under-powered, even though it did show shorter, but not statistically significant, AGDs in women with OE and DE. 

Our study found that women with adenomyosis had a shorter AGD than controls, and, in fact, shorter than those with endometriosis, suggesting that lower intrauterine androgen levels are more likely to be associated with adenomyosis, especially focal adenomyosis. This is consistent with the reports that prenatal exposure to DES increased the risk of adenomyosis in mice [61,62]. It is also in agreement, to some extent, with the evidence from animal experimentation that the neonatal feeding of estrogenic compounds such as tamoxifen or an estrogen receptor β agonist increases the risk of developing adenomyosis [63,64]. The finding that about 1/5 of the variance in AGD_AF_ could be explained by adenomyosis is also consistent with the epidemiological data including factors other than intrauterine hormonal environment, such as iatrogenic uterine procedures, increase the risk of adenomyosis [39,40,41,42,43,44], which has recently been experimentally demonstrated [45,65]. 

Our finding that no difference in AGD was found between the controls and UL patients also seems to be consistent with the known risk factors for UL, such as age, race, obesity, parity, hypertension, vitamin D deficiency, and diet in late life [11]. Of course, while prenatal DES exposure increased the risk of UL by 13% in women older than 35 years [66], another prospective cohort study employing medical records to document exposure reported no association between prenatal DES exposure and UL [67]. However, exposure during the first trimester of gestation was reported to increase the risk of UL by 21% [67]. It is possible that the effect of in utero exposure to elevated estrogen levels may be small and requires larger sample sizes to detect. Alternatively, since both estrogen and progesterone are essential for UL development [68], AGD may not contain much information on prenatal progesterone levels, and, as such, may not be a marker for UL. 

A shorter AGD in women with endometriosis and adenomyosis, but not UL, suggests a role of prenatal exposure to elevated levels of estrogen in the pathogenesis of endometriosis or adenomyosis. However, since the amount of variance that can be accounted for by the disease status is rather moderate, factors other than the prenatal hormonal exposure may also contribute to the etiology of the two diseases. In addition, many prenatal factors, unknown or yet to be identified, can also influence AGD itself. For example, prenatal exposure to stressful life events yields a longer (and thus more masculine) AGD in infant girls [69], while exposure to anti-androgens results in a shorter (and thus more feminine) AGD in infant males [23,70]. As such, the utility of AGD as a diagnostic criterion may be quite limited.

That AGD_AF_ can better discriminate patients with endometriosis from controls than AGD_AC_ has been reported previously [29], even when measured under MRI [27]. Similarly, serum testosterone levels in young women have been shown to be positively associated with a longer AGD_AF_, but not with AGD_AC_ [71]. The exact causes for this are unclear, but it is speculated to be due, in part, to the difference in the size of the fat pad anterior to the pubic *symphysis*, an area underneath the hood of the clitoris, which may have an impact on AGD_AC_ but not on AGD_AF_ [29]. If this is indeed the case, then AGD_ACt_ may also be impacted, but conceivably to a lesser extent. Consistent with this notion, we found that the correlation coefficient between the bodyweight (and BMI) and AGD measurement becomes smaller and smaller as the point of measurement goes down, presumably under the progressively falling influence of the fat pad size or simply of the bodyweight. However, that AGD_AF_ still correlated positively with the bodyweight suggests that it may be due to the bodyweight, not just the size of the fat pad anterior to the pubic *symphysis*. Thus, the positive correlation of AGD_AF_ with the bodyweight in conjunction with the negative relation with height suggests that the body fat content is associated with AGD_AF_.

Alternatively, since the point of the AGD_AC_ measurement, i.e., the anterior clitoral surface, represents the extreme end of the external genitalia while the posterior fourchette represents the other end, which is closest to the anus, the AGD_AC_ measurement contains not only the normal variation in AGD itself but also the variation in the vertical size of the genitalia. That is, the variation in AGD_AC_ has two sources: the AGD itself, and the vertical size of the genitalia. In contrast, the AGD_AF_ measurement practically does not involve the size of the external genitalia, and, as such, should be less sensitive to BMI or bodyweight, as indeed found in our data as well as in other studies such as [27]. In other words, AGD_AC_ and AGD_AF_ represent the two extremes of AGD. Thus, we argue that AGD_AF_ is the best AGD measurement as a proxy for intrauterine hormonal environment since it is insensitive to a history of vaginal delivery or of episiotomy, is least influenced by the bodyweight, and has the most discriminatory power. 

This study has several strengths. First, we analyzed the three common gynecologic diseases simultaneously. This is particularly relevant since these diseases often co-exist. More importantly, by comparing the three diseases simultaneously, we gained more insight into the difference and similarity among the diseases than investigating one single disease alone. As one Chinese adage says, “It doesn’t matter you don’t know the thing. You will know it by comparison”. Second, we added a third AGD measurement, AGD_ACt_, which sits strategically between the two known landmarks, so that we could demonstrate that AGD_AF_ is the one AGD measurement that is least affected by bodyweight while AGD_AC_ is the most affected. Third, we used three AGD measurements along with digit ratios. Lastly, we employed multiple linear regression models to control for possible confounding factors and used *R*^2^, also called coefficient of determination, to assess the contribution of each disease status to the variation in AGD. 

Our study also has several limitations. First, the controls consisted of patients who sought medical attention, and, as such, may be different from healthy controls in terms of AGD. However, most control subjects had seemingly acquired diseases, such as cervical cancer or HSIL (in which HPV infection is considered to be the greatest risk factor), making this possibility fairly remote. This seems to be borne out of the agreement with the findings as reported in [17,27,28,29,30]. Second, we did not further classify patients since endometriosis, adenomyosis, and UL each has its own classifications, such as rASRM stage, Kishi’s classification [72], and FIGO [6]. While we did document the location of endometriosis, we did not do so for adenomyosis and UL. It is possible that different subtypes of a disease may have a different etiology/pathogenesis. For example, internal adenomyosis is frequently associated with the history of uterine procedures [72]. Third, all diagnoses in this study were made based on symptoms, gynecological examinations, and imaging examination, just like other studies [28,29]. While this may still cause misclassification, we point out that it is generally accepted that imaging alone can satisfactorily diagnose endometriosis, adenomyosis, and UL [73,74,75]. In addition, any possible misclassification would only reduce the signal-to-noise ratio, obscuring the relationship between AGD and adenomyosis/endometriosis. Finally, we did not evaluate the relationship between AGD measurements in patients younger than 22 years.

## 5. Conclusions

Women with OE and adenomyosis, but not UL, were found to have significantly shorter AGD_AF_, but not AGD_AC_. OE patients, but not adenomyosis patients, had significantly shorter AGD_ACt_. In all AGD measures, AGD_AF_ is the one that has the most discriminatory power. However, the amount of variance of AGD_AF_ that could be explained by the OE and adenomyosis status is rather moderate, suggesting that factors other than the prenatal exposure to androgen/estrogen levels were also responsible for AGD. Digit ratios were unrelated to the disease status.

## Figures and Tables

**Table 1:** Characteristics among the five groups of recruited patients.

Variable	Controls (n = 100)	Endometriosis (n = 105)	Adenomyosis (n = 116)	Uterine Leiomyomas (n = 81)	Mixed (n = 28)
Age (year) *Mean ± SD* *Median (range)*	33.9 ± 6.3 34 (22–47)	34.3 ± 6.4 ^NS^ 34 (23–48)	39.5 ± 5.2 *** 39 (25–49)	39.1 ± 6.2 *** 40 (25–49)	41.5 ± 4.6 *** 41.5 (33–49)
Age at menarche (year) *Mean ± SD* *Median (range)*	13.8 ± 1.1 14(11–17)	13.3 ± 1.0 *** 13 (11–16)	13.7 ± 1.1 ^NS^ 14 (11–17)	13.5 ± 1.4 * 13 (11–17)	13.7 ± 1.0 ^NS^ 14 (12–16)
Height (m) *Mean ± SD* *Median (range)*	1.62 ± 5.7 1.61 (1.50–1.75)	1.63 ± 4.7 ^NS^ 1.63 (1.53–1.75)	1.61 ± 5.0 ^NS^ 1.60 (1.48–1.74)	1.61 ± 4.8 ^NS^ 1.60 (1.48–1.72)	1.61 ± 3.9 ^NS^ 1.62 (1.53–1.70)
Bodyweight (kg) *Mean ± SD* *Median (range)*	54.4 ± 8.5 52.5 (37.5–85)	56.2 ± 8.3 ^NS^ 55.0 (40–95)	55.5 ± 8.2 ^NS^ 55.0 (39–80)	59.0 ± 8.0 *** 58.0 (40–83)	59.1 ± 6.3 *** 58.5 (49–75)
Body mass index (BMI) (kg/m^2^) *Mean ± SD* *Median (range)*	20.7 ± 3.2 20.2(14.7–34.1)	21.2 ± 2.9 ^NS^ 20.7 (15.6–33.7)	21.5 ± 3.0 * 21.5 (16.0–30.8)	22.7 ± 2.8 *** 22.3 (17.0–32.2)	22.8 ± 2.7 *** 22.7 (18.8–29.3)
Birth weight (kg) *Mean ± SD* *Median (range)*	3.12 ± 0.35 3.0 (2.2–4.3)	3.08 ± 0.36 ^NS^ 3.1 (2.0–4.3)	3.18 ± 0.36 ^NS^ 3.1 (2.0–4.6)	3.10 ± 0.41 ^NS^ 3.0 (1.9–4.5)	3.05 ± 0.30 ^NS^ 3.0 (2.5–4.0)
Parity (n) *0* *1* *2* *≥3*	41 (41.0%) 41 (41.0%) 15 (15.0%) 3 (3.0%)	40 (38.1%) ^NS^ 55 (52.4%) 9 (8.6%) 1 (1.0%)	17 (14.7%) *** 80 (69.0%) 16 (13.8%) 3 (2.6%)	24 (29.6%) ^NS^ 45 (55.6%) 10 (12.3%) 2 (2.5%)	6 (21.4%) ^NS^ 17 (60.7%) 5 (17.9%) 0 (0.0%)
Vaginal deliveries *0* *1* *2* *≥3*	59 (59.0%) 34 (34.0%) 6 (6.0%) 1 (1.0%)	70 (66.7%) ^NS^ 33 (31.4%) 1 (1.0%) 1 (1.0%)	65 (56.0%) ^NS^ 44 (37.9%) 5 (4.3%) 2 (1.7%)	46 (56.8%) ^NS^ 31 (38.3%) 3 (3.7%) 1 (1.2%)	17 (60.7%) ^NS^ 11 (39.3%) 0 (0.0%) 0 (0.0%)
Episiotomy *No* *Yes*	73 (73.0%) 27 (27.0%)	60 (74.1%) ^NS^ 21 (25.9%)	75 (71.4%) ^NS^ 30 (28.6%)	77 (66.4%) ^NS^ 39 (33.6%)	17 (60.7%) ^NS^ 11 (39.3%)
Intrauterine surgeries (n) *0* *1* *≥2*	55 (55.0%) 26 (26.0%) 19 (19.0%)	66(64.8%) * 32 (30.5%) 7 (6.7%)	46 (39.7%) ^NS^ 36 (31.0%) 34 (29.3%)	31 (38.3%) ^NS^ 26 (32.1%) 24 (29.6%)	8 (28.6%) * 9 (32.1%) 11 (39.3%)
Cesarean section *0* *1* *≥2*	80 (80.0%) 13 (13.0%) 7 (7.0%)	68 (64.8%) ** 30 (25.6%) 7 (6.7%)	66 (56.9%) *** 46 (38.8%) 5 (4.3%)	57 (70.4%) ^NS^ 21 (25.9%) 3 (3.7%)	16 (57.1%) * 9 (32.1%) 3 (10.7%)
Severity of dysmenorrhea *None* *Mild* *Moderate Severe*	99 (99.0%) 1 (1.0%) 0 (0.0%) 0 (0.0%)	9 (8.6%) *** 42 (40.0%) 23 (21.9%) 31 (29.5%)	4 (3.4%) *** 12 (10.3%) 31 (26.7%) 69 (59.5%)	72 (88.9%) ** 9 (11.1%) 0 (0.0%) 0 (0.0%)	0 (0.0%) *** 5 (17.9%) 6 (21.4%) 17 (60.7%)
Co-occurrence with ovarian endometrioma *No* *Yes*	100 (100.0%) 0 (0.0%)	5 (4.8%) 100 (95.2%)	110 (94.8%) 6 (5.2%)	81 (100.0%) 0 (0.0%)	24 (85.7%) 4 (14.3%)
Co-occurrence with pelvic endometriosis *No* *Yes*	100 (100.0%) 0 (0.0%)	104 (99.0%) 1 (1.0%)	116 (0.0%) 0 (0.0%)	81 (100.0%) 0 (0.0%)	28 (100.0%) 0 (0.0%)
Co-occurrence with deep endometriosis *No* *Yes*	100 (100.0%) 0 (0.0%)	78 (72.4%) 29 (27.6%)	112 (96.6%) 4 (3.4%)	81 (100.0%) 0 (0.0%)	24 (85.7%) 4 (14.3%)
Co-occurrence with other types of endometriosis *No* *Yes*	100 (100.0%) 0 (0.0%)	100 (95.2%) 5 (4.8%)	116 (0.0%) 0 (0.0%)	81 (100.0%) 0 (0.0%)	28 (100.0%) 0 (0.0%)
Co-occurrence with adenomyosis *No* *Diffuse* *Focal*	100 (100.0%) 0 (0.0%) 0 (0.0%)	81 (77.1%) 14 (13.3%) 10 (9.5%)	0 (0.0%) 90 (77.6%) 26 (22.4%)	81 (100.0%) 0 (0.0%) 0 (0.0%)	0 (0.0%) 14 (50.0%) 14 (50.0%)
Co-occurrence with uterine leiomyomas *No* *Yes*	100 (100.0%) 0 (0.0%)	88 (83.8%) 17 (16.2%)	114 (98.3%) 2 (1.7%)	0 (0.0%) 81 (100.0%)	0 (0.0%) 28 (100.0%)

*: *p* < 0.05; **: *p* < 0.01; ***: *p* < 0.001; ^NS^: not significant, i.e., *p* > 0.05.

**Table 2:** Summary of anthropometric biomarker measurements in different patient groups.

Biomarker	Control	Endometriosis	Adenomyosis	Uterine Leiomyomas	Mixed
AGD Measures					
AGD_AC_ (mm) Mean ± S.D. 25th percentile Median 75% percentile	97.6 ± 8.2 92 99 102.3	97.4 ± 8.5 ^NS^ 92 98 102	99.3 ± 9.2 ^NS^ 94 100.4 104	99.9 ± 10.3 * 95 101.6 106	100.1 ± 9.9 ^NS^ 95 100 103.5
AgD_ACt_ (mm) Mean ± S.D. 25th percentile Median 75% percentile	73.4 ± 8.0 68 72 78	72.2 ± 8.9 ^NS^ 66 71 78	73.6 ± 8.2 ^NS^ 69.8 73 80	74.5 ± 9.5 ^NS^ 67 76 80.5	74.6 ± 9.0 ^NS^ 67.4 74.5 82
AGD_AF_ (mm) Mean ± S.D. 25th percentile Median 75% percentile	25.1 ± 4.0 22 24.5 28	21.5 ± 3.1 *** 20 21.9 23	20.8 ± 2.9 *** 19 21 22.5	24.6 ± 4.2 ^NS^ 22 24 27	21.7 ± 2.1 *** 20.9 21 23
**Digit ratio**					
Left-hand Mean ± S.D. 25th percentile Median 75% percentile	0.972 ± 0.029 0.954 0.972 0.986 (Missing: n = 1)	^NS^ 0.977 ± 0.032 0.957 0.973 0.993	* 0.981 ± 0.031 0.958 0.984 1.000	^NS^ 0.973 ± 0.031 0.953 0.972 0.986	^NS^ 0.976 ± 0.040 0.953 0.978 1.004
Right-hand Mean ± S.D. 25th percentile Median 75% percentile	0.977 ± 0.027 0.958 0.973 1.000 (Missing: n = 1)	^NS^ 0.975 ± 0.028 0.960 0.971 0.992 (Missing: n = 1)	^NS^ 0.978 ± 0.034 0.956 0.978 1.000 (Missing: n = 1)	^NS^ 0.972 ± 0.034 0.953 0.970 0.985	^NS^ 0.974 ± 0.037 0.951 0.972 1.000

***: *p* < 0.001; *: *p* < 0.05; ^NS^: not significant, i.e., *p* > 0.05.

## Data Availability

The data presented in this study are available upon written request from the corresponding author explaining the use and purposes.

## Supplementary Materials

The following supporting information can be downloaded at: https://www.mdpi.com/article/10.3390/biomedicines11102618/s1, Figure S1: Distribution of different patient characteristics in different groups. Boxplot showing the distribution of age (A), age at menarche (B), birthweight (C), height (D), bodyweight (E), and body mass index (BMI) (F). In all plots, the statistical comparison was made between the designated group and the control group using the Wilcoxon’s test. Symbol for statistical significance level: NS: *p* > 0.05; *: *p* < 0.05; ***: *p* < 0.001.
