# Supplementary material for: Shorter Anogenital Distance in Women with Ovarian Endometriomas and Adenomyosis, but Not Uterine Leiomyomas
Source: Biomedicines. 2023 Sep 23;11(10):2618. doi: 10.3390/biomedicines11102618 (PMC10603971; doi:10.3390/biomedicines11102618)
Supplement: Supplementary file 1 [file biomedicines-11-02618-s001.zip › biomedicines-2576281-supplementary.pdf]

Supplementary materials

Shorter Anogenital Distance in Women with Ovarian Endometriomas and Adenomyosis, But Not Uterine Leiomyomas

Xishi Liu, Ding Ding, Minhong Shen, Dingmin Yan and Sun-Wei Guo

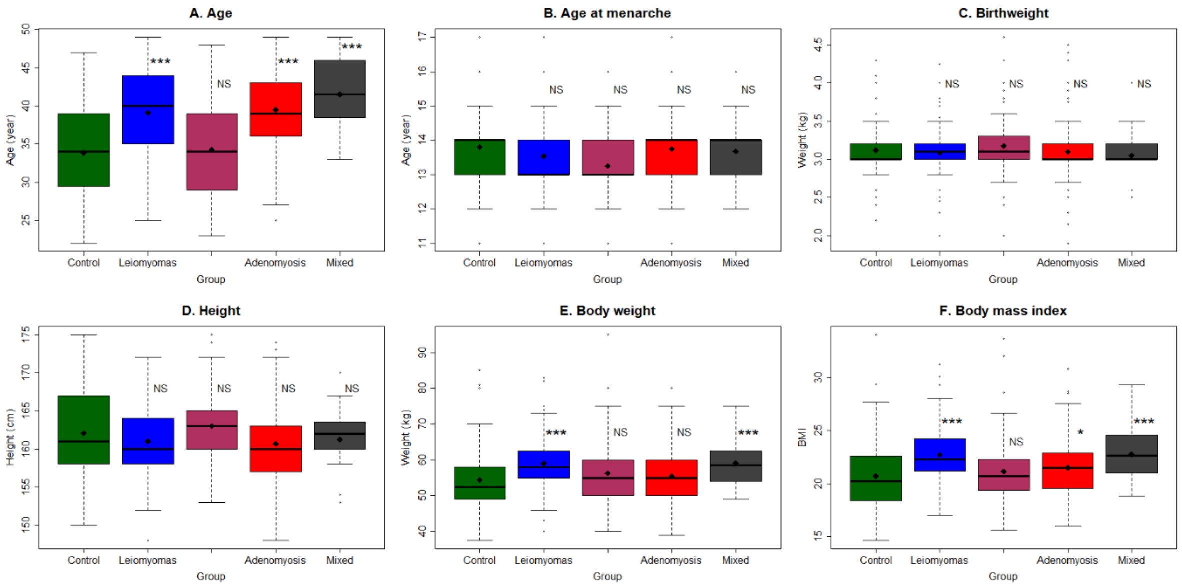

Supplementary Figure S1. Distribution of different patient characteristics in different groups. Boxplot showing the distribution of age (A), age at menarche (B), birthweight (C), height (D), body weight (E), and body mass index (BMI) (F). In all plots, the statistical comparison was made between the designated group and the Control group, using the Wilcoxon's test. Symbol for statistical significance level: NS: p>0.05; *: p<0.05; ***: p<0.001.
